# Supplementary material for: Unveiling the Bioactive Potential of the Invasive Jellyfish Phyllorhiza punctata Through Integrative Transcriptomic and Proteomic Analyses
Source: Biomolecules. 2025 Aug 4;15(8):1121. doi: 10.3390/biom15081121 (PMC12383608; doi:10.3390/biom15081121)
Supplement: Supplementary file 1 [file biomolecules-15-01121-s001.zip › Suplementary Material/FileS3/PEPFOLD4/XP_065053426_11083/clusters.html]

 PEP-FOLD clusters


Download archive of all models  here   

|  |  |  |
| --- | --- | --- |
| # | file | sOPEP |
| # cluster 1 - Size: 4 |  |  |
|  | XP\_065053426\_11083-model1 | -26.6403 |
|  | XP\_065053426\_11083-model1.1 | -26.52 |
|  | XP\_065053426\_11083-model1.2 | -26.4904 |
|  | XP\_065053426\_11083-model1.3 | -26.4137 |
| # cluster 2 - Size: 15 |  |  |
|  | XP\_065053426\_11083-model2 | -23.2382 |
|  | XP\_065053426\_11083-model2.1 | -23.0831 |
|  | XP\_065053426\_11083-model2.2 | -23.0003 |
|  | XP\_065053426\_11083-model2.3 | -22.9906 |
|  | XP\_065053426\_11083-model2.4 | -22.9678 |
|  | XP\_065053426\_11083-model2.5 | -22.9207 |
|  | XP\_065053426\_11083-model2.6 | -22.8701 |
|  | XP\_065053426\_11083-model2.7 | -22.8487 |
|  | XP\_065053426\_11083-model2.8 | -22.7915 |
|  | XP\_065053426\_11083-model2.9 | -22.768 |
|  | XP\_065053426\_11083-model2.10 | -22.6681 |
|  | XP\_065053426\_11083-model2.11 | -22.6608 |
|  | XP\_065053426\_11083-model2.12 | -22.4905 |
|  | XP\_065053426\_11083-model2.13 | -22.4649 |
|  | XP\_065053426\_11083-model2.14 | -22.4051 |
| # cluster 3 - Size: 22 |  |  |
|  | XP\_065053426\_11083-model3 | -23.1888 |
|  | XP\_065053426\_11083-model3.1 | -23.1468 |
|  | XP\_065053426\_11083-model3.2 | -23.1125 |
|  | XP\_065053426\_11083-model3.3 | -22.9543 |
|  | XP\_065053426\_11083-model3.4 | -22.9522 |
|  | XP\_065053426\_11083-model3.5 | -22.9341 |
|  | XP\_065053426\_11083-model3.6 | -22.8905 |
|  | XP\_065053426\_11083-model3.7 | -22.8383 |
|  | XP\_065053426\_11083-model3.8 | -22.8047 |
|  | XP\_065053426\_11083-model3.9 | -22.7938 |
|  | XP\_065053426\_11083-model3.10 | -22.7513 |
|  | XP\_065053426\_11083-model3.11 | -22.7268 |
|  | XP\_065053426\_11083-model3.12 | -22.7258 |
|  | XP\_065053426\_11083-model3.13 | -22.7253 |
|  | XP\_065053426\_11083-model3.14 | -22.7235 |
|  | XP\_065053426\_11083-model3.15 | -22.7207 |
|  | XP\_065053426\_11083-model3.16 | -22.695 |
|  | XP\_065053426\_11083-model3.17 | -22.6918 |
|  | XP\_065053426\_11083-model3.18 | -22.6436 |
|  | XP\_065053426\_11083-model3.19 | -22.6233 |
|  | XP\_065053426\_11083-model3.20 | -22.4563 |
|  | XP\_065053426\_11083-model3.21 | -22.3487 |
| # cluster 4 - Size: 7 |  |  |
|  | XP\_065053426\_11083-model4 | -23.0765 |
|  | XP\_065053426\_11083-model4.1 | -23.0057 |
|  | XP\_065053426\_11083-model4.2 | -22.8837 |
|  | XP\_065053426\_11083-model4.3 | -22.805 |
|  | XP\_065053426\_11083-model4.4 | -22.7721 |
|  | XP\_065053426\_11083-model4.5 | -22.7096 |
|  | XP\_065053426\_11083-model4.6 | -22.1075 |
| # cluster 5 - Size: 14 |  |  |
|  | XP\_065053426\_11083-model5 | -23.0714 |
|  | XP\_065053426\_11083-model5.1 | -22.9939 |
|  | XP\_065053426\_11083-model5.2 | -22.9792 |
|  | XP\_065053426\_11083-model5.3 | -22.9721 |
|  | XP\_065053426\_11083-model5.4 | -22.9579 |
|  | XP\_065053426\_11083-model5.5 | -22.9354 |
|  | XP\_065053426\_11083-model5.6 | -22.8939 |
|  | XP\_065053426\_11083-model5.7 | -22.8405 |
|  | XP\_065053426\_11083-model5.8 | -22.8062 |
|  | XP\_065053426\_11083-model5.9 | -22.7949 |
|  | XP\_065053426\_11083-model5.10 | -22.7492 |
|  | XP\_065053426\_11083-model5.11 | -22.6689 |
|  | XP\_065053426\_11083-model5.12 | -22.617 |
|  | XP\_065053426\_11083-model5.13 | -22.2706 |
| # cluster 6 - Size: 1 |  |  |
|  | XP\_065053426\_11083-model6 | -22.9438 |
| # cluster 7 - Size: 3 |  |  |
|  | XP\_065053426\_11083-model7 | -22.913 |
|  | XP\_065053426\_11083-model7.1 | -22.9092 |
|  | XP\_065053426\_11083-model7.2 | -22.5027 |
| # cluster 8 - Size: 14 |  |  |
|  | XP\_065053426\_11083-model8 | -22.8599 |
|  | XP\_065053426\_11083-model8.1 | -22.8441 |
|  | XP\_065053426\_11083-model8.2 | -22.8035 |
|  | XP\_065053426\_11083-model8.3 | -22.803 |
|  | XP\_065053426\_11083-model8.4 | -22.7837 |
|  | XP\_065053426\_11083-model8.5 | -22.776 |
|  | XP\_065053426\_11083-model8.6 | -22.6414 |
|  | XP\_065053426\_11083-model8.7 | -22.6285 |
|  | XP\_065053426\_11083-model8.8 | -22.6137 |
|  | XP\_065053426\_11083-model8.9 | -22.5814 |
|  | XP\_065053426\_11083-model8.10 | -22.5294 |
|  | XP\_065053426\_11083-model8.11 | -22.4699 |
|  | XP\_065053426\_11083-model8.12 | -22.4074 |
|  | XP\_065053426\_11083-model8.13 | -21.9431 |
| # cluster 9 - Size: 7 |  |  |
|  | XP\_065053426\_11083-model9 | -22.8013 |
|  | XP\_065053426\_11083-model9.1 | -22.7877 |
|  | XP\_065053426\_11083-model9.2 | -22.7863 |
|  | XP\_065053426\_11083-model9.3 | -22.6637 |
|  | XP\_065053426\_11083-model9.4 | -22.6516 |
|  | XP\_065053426\_11083-model9.5 | -22.5618 |
|  | XP\_065053426\_11083-model9.6 | -22.4849 |
| # cluster 10 - Size: 4 |  |  |
|  | XP\_065053426\_11083-model10 | -15.7425 |
|  | XP\_065053426\_11083-model10.1 | -15.5406 |
|  | XP\_065053426\_11083-model10.2 | -15.2098 |
|  | XP\_065053426\_11083-model10.3 | -14.8336 |
| # cluster 11 - Size: 4 |  |  |
|  | XP\_065053426\_11083-model11 | -10.8292 |
|  | XP\_065053426\_11083-model11.1 | -10.7813 |
|  | XP\_065053426\_11083-model11.2 | -10.4338 |
|  | XP\_065053426\_11083-model11.3 | -9.45005 |
| # cluster 12 - Size: 1 |  |  |
|  | XP\_065053426\_11083-model12 | -10.6876 |
| # cluster 13 - Size: 1 |  |  |
|  | XP\_065053426\_11083-model13 | -10.6114 |
| # cluster 14 - Size: 2 |  |  |
|  | XP\_065053426\_11083-model14 | -9.88025 |
|  | XP\_065053426\_11083-model14.1 | -9.2142 |
| # cluster 15 - Size: 1 |  |  |
|  | XP\_065053426\_11083-model15 | -9.82831 |
|  |  |  |
